# Treatment retention in opioid agonist therapy: comparison of methadone versus buprenorphine/naloxone by analysis of daily-witnessed dispensed medication in a Canadian Province

**DOI:** 10.1186/s12888-022-04175-9

**Published:** 2022-07-30

**Authors:** Joseph Sadek, Joseph Saunders

**Affiliations:** 1 Department of Psychiatry, Dalhousie University, 810 Maplewood Lane, Halifax, NS B3H 4k3 Canada; 2 Department of Medicine, Dalhousie University, Halifax, NS Canada

**Keywords:** Opioid use, Buprenorphine/naloxone, Methadone, Treatment retention

## Abstract

**Background:**

The last decade has shown a remarkable increase in the rates of illicit opioid use in Canada and internationally, which is associated with large increases in opioid related morbidity and mortality. While the differences between methadone and buprenorphine/naloxone in terms of retention have been studied outside Canada, the unique location and design of this study, gives it a specific significance.

**Objectives:**

This study aims to describe the relative treatment retention rates for first episode opioid replacement treatment between methadone and buprenorphine/naloxone for patients receiving daily witnessed dispensed medications in Nova Scotia.

**Methods:**

A longitudinal retrospective descriptive study analyzing secondary data from the Nova Scotia Prescription Monitoring Program on patients 18 years of age and older who started first episode opioid agonist therapy with methadone or buprenorphine/naloxone for opioid use disorder in Nova Scotia between 2014 and 2018. Treatment episode was defined as date of initial opioid agonist prescription until there is a gap of greater than 6 days without receiving opioid agonist medication at a pharmacy.

**Results:**

One thousand eight hundred sixty-seven of whom were analyzed as they had at least 1 day in treatment. There was significant treatment dropout within the first 2 weeks of treatment, which did not show a significant difference between OAT medication (23.4% of buprenorphine/naloxone; 22.2% methadone). Median duration of retention in treatment was 58 days for those treated with buprenorphine/naloxone and 101 days for patients treated with methadone.

Multivariate cox proportional hazards model showed that buprenorphine/naloxone use as compared to methadone lead to increased hazard of treatment dropout by 62% (HR = 1.62).

Hazard rate of treatment dropout for patients below 25 years of age was calculated. (HR 1.53).

Median duration of retention in treatment for this subgroup of patients younger than age 25 was 37.5 days for patients treated with buprenorphine/naloxone and 69 days for patients treated with methadone.

**Conclusions:**

Our data suggests that methadone is a numerically superior medication for opioid use disorder when the metric of treatment retention is viewed in isolation, for our population in Nova Scotia. However, the results should be interpreted carefully considering the number of limitations of this study. There are social/accessibility, pharmacologic/safety, and patient preference factors which are also key in decision making when prescribing opioid agonist therapy. These must all be considered when deciding on which medication to initiate for a patient beginning a new treatment episode with OAT for opioid use disorder. This study should stimulate further research into this important area in addiction medicine.

## Background

The last decade has shown a remarkable increase in the rates of non-medical prescription and illicit opioid use in Canada and internationally, which is associated with large increases in opioid related morbidity and mortality [1, 2]. Opioid use disorder (OUD), described as a pattern of opioid use that is chronic and interferes with function. It is a major contributor to serious public health concerns [3, 4]. Injection drug use has increased concomitantly with the increasing rates of non-medical opioid use, leading to new outbreaks of HCV and HIV that have disproportionally affected rural areas in Canada [5, 6].

### Treatment models and strategies

Standard of care treatment of OUD is pharmacological opioid replacement with opioid agonists such as methadone and buprenorphine [7]. Buprenorphine is commonly prescribed in Canada in combination with naloxone, an opioid antagonist used to mitigate risk of overdose if the combination is taken intravenously, as buprenorphine/naloxone (Suboxone) and to reduce the risk of abuse and diversion [5]. The benefits of opioid agonist therapy (OAT) have been very well described and consistently show decreased morbidity and mortality, decreased rates of criminal justice interaction, and decreased rates of bloodborne disease transmission [8].

The delivery of OAT varies greatly between countries and even regionally across Canada. In the United States, methadone treatment is delivered in the setting of opioid treatment programs (OTPs), where patients visit a designated site daily to receive OAT, while buprenorphine is delivered more flexibly by primary care physician prescription and dispensed at local pharmacies [7]. In Canada, both methadone and buprenorphine/naloxone are provided through designated opioid treatment programs (provincially and privately run, depending on the jurisdiction), and through direct access to primary care physicians, with medication being dispensed at community pharmacies [7, 8]. At the community pharmacy patients may either receive medication dispensed daily with doses witnessed by the pharmacist, or as “take-home” doses where patients are dispensed medication to take with them, with a quantity dispensed to last for several days to several weeks.

Treatment retention is a critical outcome when deciding on appropriate medication for patients with opioid use disorders, as rates of relapse and opioid related mortality are vastly increased on cessation of treatment [9]. A recent Cochrane meta-analysis of retention rates in studies comparing treatment retention in methadone and buprenorphine demonstrated that for flexible dosing (ie. titration of dose to that required for cessation of withdrawal symptoms in patients) of both medications, which is most reflective of clinical practice, methadone continues to show significantly greater retention rates [10]. The safety profile of buprenorphine, however, makes this medication much more attractive for treating opioid use disorder in the office setting where there is less control over the medication and fewer opportunities to observe patients [8].

### Current climate in Canada

Recent Canadian practice guidelines for the treatment of OUD suggest that buprenorphine/naloxone should be recommended as first line for OAT in most people [4]. The guidelines suggest that buprenorphine/naloxone is advantageous because it is relatively safe to dispense as a take home medication early in treatment (within 7–10 days of initiation), which has the potential to tremendously impact the lifestyle of patients. However, this strategy is used inconsistently across Canada. While in British Columbia there are many ongoing initiatives to further decrease barriers to treatment access, including rapid access to take home doses and hydromorphone and diacetylmorphine prescription is becoming more standard, there are still conventions in other provinces, including Nova Scotia, which limit take home doses to patients who have already been retained in treatment for significant periods of time (often 3 months or greater) [11]. Between 2011 and 2017 there has been an increase from 2062 to 4088 patients dispensed methadone or buprenorphine/naloxone in Nova Scotia, suggesting that further studies of treatment outcomes in our population are increasingly relevant [12].

There have been few epidemiological studies of opioid replacement retention rates in the Canadian context, and none that we know of in Nova Scotia. While the differences between methadone and buprenorphine/naloxone in terms of retention have been well studied, we wish to describe the data in the Nova Scotian context of current prescribing patterns favouring community pharmacy dispensing of daily witnessed medication.

This study was intended to provide a contemporary description of the treatment retention landscape of OAT in Nova Scotia to allow for greater insight into what is currently working in the context of primarily daily witnessed dispensation of methadone and buprenorphine/naloxone, and to consider what may be gained by implementing policies that have been successful in other jurisdictions.

## Methods

This study aims to describe the relative treatment retention rates for first episode opioid replacement treatment between methadone and buprenorphine/naloxone for patients receiving daily witnessed dispensed medications in Nova Scotia.

It is a longitudinal retrospective descriptive study analysing secondary data from the Nova Scotia Prescription Monitoring Program (NSPMP). This data were collected in the process of the NSPMP’s mandate to monitor prescribing and dispensing information of all controlled substances in Nova Scotia at community pharmacies.

All data were delivered to researchers in a de-identified format. No PHI was held by researchers at any point in this study. All study data was kept secure as per NSHA IT policy.

### Study population

The study population included all patients aged 18 and older who were eligible for provincial health benefits and had initiated a first treatment episode of opioid agonist therapy in Nova Scotia between January 1, 2014 and December 31st, 2018. A treatment episode was defined as date of initial opioid agonist prescription until there is a gap of greater than 6 days without receiving opioid agonist medication at a pharmacy.

Patients were excluded from the study analysis if they switched from the opioid agonist on which they began treatment, such as initiating on methadone and subsequently switching to buprenorphine-naloxone or slow-release oral morphine. Patients who were maintained on opioid agonists other than methadone and buprenorphine-naloxone were also excluded.

For the study population, patients prescribed buprenorphine during the relevant treatment episode were 36.49% females and 63.51% males. Patients prescribed methadone during the relevant treatment episode were 37.98% female and 62.02% male. Overall, 37.70% of the study population was female and 62.30% of the study population was male.

### Data extraction and analysis

Data extracted from the NSPMP electronic database was de-identified when delivered to researchers and was stored in accordance with NSHA protocol.

Requested patient variables were:

- Gender
- Age (stratified in groups 18–19, 20–24, 25–29, 30–39, 40–49, 50–59, 60+)
- Date of initiation of first treatment episode
- Date of end of first treatment episode
- Number of days where daily dispensed medications were not picked up during treatment episode.
- Opioid agonist medication prescribed (methadone or buprenorphine-naloxone)
- History of psychostimulant prescriptions at Nova Scotia community pharmacy (Yes/No)

Both initial treatment episode during study period and subsequent treatment episodes meeting inclusion criteria were assessed.

Data analysis was conducted using STATA version 13 and Microsoft Excel. We compared treatment retention between methadone and buprenorphine/naloxone using Kaplan-Meier survival analysis for categorical variables as described above. A cox proportional hazard model was used to characterize the risk of treatment dropout based on a final model of significant univariate predictors. A significance level of 0.05 was employed in the relevant analyses.

In interpretation of this data, it is important to recognize that the NSPMP is primarily concerned with monitoring-controlled prescription medication dispensing information and is not involved in the diagnostic process, thus we do not have information directly linking patients to a diagnosis of opioid use disorder. However, most patients who are prescribed methadone or buprenorphine/naloxone receive the prescription for the purpose of opioid replacement, and furthermore, those who are prescribed daily witnessed doses are virtually exclusively taking the medication in the context of opioid replacement.

The study authors did not have access to any data as to decision making around patients receiving take home doses. Take home doses were determined by pharmacy reporting of number of doses dispensed on a given day. While patients receiving daily witnessed dispensed medications would receive a quantity of “1” per day, some days such as holidays and store closures necessitate a limited number of take-home doses for patients who were still engaged in intensive treatment. Therefore, authors decided that patients who did not at any time receive more than 3 doses dispensed on a given day would be considered not to have consistent take home doses, and thus allowed inclusion in the analysis. This was on the rationale of most patients receiving consistent take home doses progressing on a graduated schedule of bi-weekly take home doses (3 days + 4 days) and then weekly (7 days) supplies.

Research Ethics approval for this work was sought and was granted by the Nova Scotia Health Authority Research Ethics Board.

## Results

Demographic information for the population analysed is detailed in Table 1. Most patients were younger than age 40, used methadone, and did not have a history of medical psychostimulant use. The population consisted of 1886 patients who began their first treatment with opioid agonist therapy between January 1, 2014, and December 31, 2018 who received only daily witnessed medication (never more than 2 take home doses at a time during the treatment episode).

**Table 1:** Study population characteristics, by age group, medication type, stimulant use history, sex

Age	Freq.	%	Cum. %	OAT Medication		Stimulant use hx		Sex	
				Methadone	Bup/Nx	No	Yes	M	F
18–19	41	2.17	2.17	22	19	27	14	16	25
20–24	320	16.97	19.14	241	79	238	82	162	158
25–29	432	22.91	42.05	359	73	314	118	248	184
30–34	333	17.66	59.70	274	59	236	97	216	117
35–39	282	14.95	74.66	230	52	212	70	183	99
40–44	181	9.60	84.25	148	33	148	33	121	60
45–49	119	6.31	90.56	102	17	98	21	88	31
50–54	95	5.04	95.60	85	10	89	6	76	19
55–59	56	2.97	98.57	44	12	52	4	46	10
60+	27	1.43	100	22	5	24	3	19	8
Total	1886	100		1527 (81%)	359 (19%)	1438	448	1175	711

The proportions of each opioid agonist medication used varied over years covered by study period, with proportion of buprenorphine/naloxone used increasing from 3% in 2014 to 50.5% in 2018. Detailed OAT use by year is shown in Table 2.

**Table 2:** OAT Medication breakdown, number of patients, by year of study period

Year	Methadone	Buprenorphine/Nx
2014	350	11
2015	393	37
2016	304	36
2017	291	82
2018	189	193

Individuals who had fewer than 1 complete days of treatment were excluded from further analysis, leaving 1867 patients.

Univariate analysis was conducted for categorical predictors of treatment retention including OAT medication, age groupings, sex, and previous history of medical prescription psychostimulant use.

OAT medication and previous history of medical prescription psychostimulant use were significant for equality across strata and thus were considered for possible inclusion in multivariate models. Sex and age groupings were not significant predictors of treatment retention in this sample.

A multivariate Cox proportional hazards model was created using OAT medication and previous history of medical prescription psychostimulant use. After accounting for previous history of medical prescription psychostimulant use, buprenorphine-naloxone use as compared to methadone lead to increased hazard rate of treatment dropout by 62% (HR = 1.62). History of medical prescription psychostimulant use led to an increase in hazard rate of treatment dropout by 27% (HR 1.27) after accounting for OAT medication. There were no significant interactions between predictor variables and thus these were not included in the final model.

The proportional hazards assumption test was conducted, showing Prob> χ^2^ = 0.261, leading us to fail to reject the null hypothesis that the hazards are proportional for this model.

There was significant treatment dropout within the first 2 weeks in our sample; 85 (23.4%) patients on buprenorphine/naloxone and 339 (22.2%) patients on methadone ended treatment within the first 14 days.

**Fig. 1:**
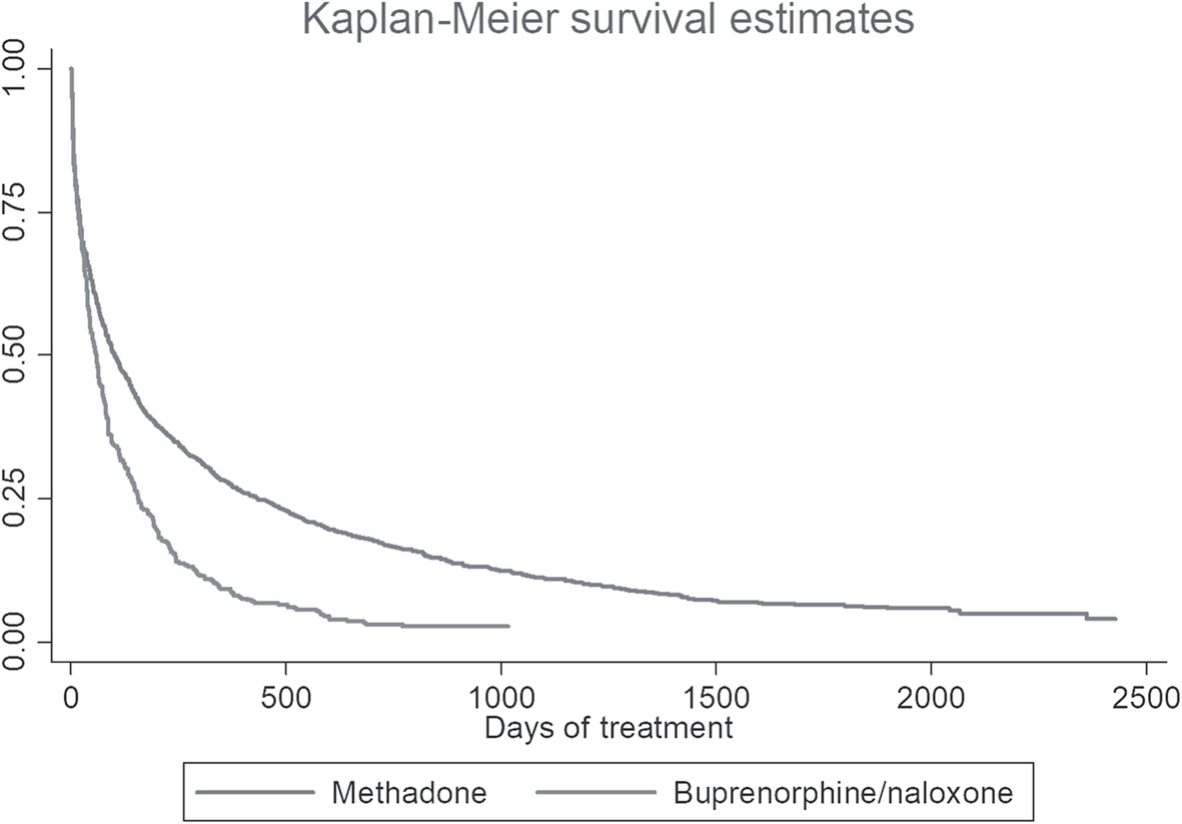
Kaplan-Meier survival estimates for treatment retention by OAT medication (buprenorphine/naloxone vs methadone). Median duration of retention in treatment was 58 days for those treated with buprenorphine/naloxone and 101 days for patients treated with methadone

Median duration of retention in treatment was 58 days for those treated with buprenorphine/naloxone and 101 days for patients treated with methadone (Fig. 1).

Subgroup analysis was conducted for patients younger than age 25. Univariate Cox proportional hazards model was created using OAT medication groups, which showed buprenorphine-naloxone use as compared to methadone lead to increased hazard rate of treatment dropout by 53% (HR 1.53).

Median duration of retention in treatment for this subgroup of patients younger than age 25 was 37.5 days for patients treated with buprenorphine/naloxone and 69 days for patients treated with methadone.

## Discussion

Our data continue to provide insight into the relationship between medication for opioid use disorders and retention in treatment for the subset of patients who receive predominantly daily witnessed medications. These patients are typically those who are less stable, and clinically represent most patients on long term medications for opioid use disorder.

In agreement with the Cochrane meta-analysis from 2014 [10], we found that patients maintained on buprenorphine/naloxone were less likely to be retained in treatment at a given time after induction than those started on methadone. In the meta-analysis, this finding was for “flexible dosing” of buprenorphine, which is the typical clinical practice – continued titration until enough protection is offered from withdrawal symptoms.

We also found that the initial retention in the first several weeks of therapy was quite similar between the medications, with an insignificant difference between methadone and buprenorphine/naloxone groups on dropout in the first 14 days. In our sample we report fewer drop-outs in the first 2 weeks than is typically shown in other epidemiological samples of OAT retention [13, 14],

Many strategies have been described to improve treatment retention amongst patients enrolled in OAT, including alternative means of care delivery, integration of multiple medical/psychiatric/social services, contingency management/reward systems, and long acting formulations of OAT medications [15]. While treatment retention as an outcome was not significantly different between many of these strategies, a recent study from Ontario, Canada described greater retention when care was offered in a telehealth/virtual care model [16].

The study period covered treatment initiated between 2014 and 2018 and follow up continued through September 2020. The treatment landscape changed significantly with respect to prescribing practices in Nova Scotia, and this is reflected in our data for this specific population. In 2014, only 3% of OAT consisted of buprenorphine, while in 2018 it comprised 50% of total OAT medication prescriptions in our population. This shift likely represents a provider preference toward initiating patients on buprenorphine/naloxone as it has less risk crushing and injecting buprenorphine and a more tolerable safety profile, particularly in the context of the trend toward more permissive and rapid access to “take home” dosing of OAT medications [17].

Yet another emerging technique to improve treatment retention, and which may be contributing to the shift of prescribing further towards buprenorphine/naloxone, is the practice of micro-induction dosing of this medication, such that patients can be initiated on buprenorphine/naloxone concurrently with ongoing full opioid agonists and titrated to a treatment dose prior to cessation of full agonist medication such that opioid withdrawal is avoided [17, 18].

A secondary outcome of interest is the potential association of prescription psychostimulant use history on treatment retention in this population. We used this indicator as a proxy for past diagnosis and treatment of Attention Deficit Hyperactivity Disorder (ADHD), although we did not have information on current psychostimulant use or compliance with this medication category. We found that a positive history of this indicator was associated with decreased treatment retention at any given time, after accounting for the opioid agonist medication that the patient was prescribed. ADHD is a well documented risk factor predisposing to substance use disorders [19], and it has previously been described that ADHD may be a factor leading to decreased retention in treatment for use with medications for opioid use disorder [20, 21].

Further subgroup analysis on patients under age 25 was conducted as this is a particularly vulnerable population in terms of morbidity and mortality associated with opioid use disorder [22]. A position statement by the American Academy of Pediatrics has been published recently which suggests the paramount importance of engaging this population and offering the most evidence based MOUD treatment options as first line [23]. Our analysis suggests that methadone continues to offer a superior profile of treatment retention as compared to buprenorphine/naloxone in this age group, despite a changing landscape of treatment in recent years.

Interestingly, the association between history of psychostimulant prescription and decreased treatment retention was not maintained in the subgroup analysis of patients younger than 25, such that there was no difference in treatment retention by positive history of psychostimulant prescription. This may be reflective of a previously proposed theory of early onset prescribing of ADHD medication acting as a protective factor against ADHD related difficulties with complying with treatment, though more research will be needed [24].

There are several clear limitations to the current study. We were using data collected for the purpose of prescription monitoring and not specifically for epidemiological research. There were several difficulties in translating this data to make clinically relevant conclusions, including a limited number of instances where pharmacies reported that patients had received doses when in fact they had not, as they may occasionally bill ahead for OAT medications for an entire day regardless of when a patient picks up their dose. There was the potential that several patients may have been inappropriately excluded because they may have been marked as having take home doses (dispensed > 3 days sequential medication) when it was in fact a pharmacy transcription error. Previous history of psychostimulant use as a proxy for ADHD is an imperfect measure and does not give us any information about the validity of the diagnosis or the ongoing medical treatment of it. Finally, the COVID-19 pandemic began during the follow up window for treatment monitoring, and as such some patients may have received take home doses of medication while otherwise falling into the population we were trying to characterize, potentially leading to premature stop dates of treatment episodes.

Choice of OAT medication for each treatment episode was a prescriber-specific decision. It was made in discussion with the patient based on previous experiences of both the prescriber and patient, as well as the individual clinical profile of the patient.

The data are collected from community pharmacies, and they do not represent any prescribing patterns in hospital or institutional settings. The data for this study period is intended to represent maintenance dosage of each opioid agonist. It is prohibited to send patients to specific pharmacy in NS therefore numerous pharmacies were used to dispense OAT. Evaluation of site differences was beyond the scope of this analysis of secondary data, but.

this could be useful research In the future.

Over the last 18 months, the COVID-19 pandemic has drastically shifted the practices of prescribers worldwide with respect to OAT. Rapid adoption of tele-health services and loosening of restrictions on “take home” doses were adopted in many countries [25, 26]. While this is still a very active area of research, these changes were seen as a public health imperative both to decrease transmission of COVID-19 and to attempt to mitigate the social and environmental pressures of the pandemic on already vulnerable populations [27]. This shift in OAT practice will undoubtedly have an impact on prospective rates of treatment retention, and there will be ample need for follow up study of these trends.

There are many variables which contribute to treatment initiation and dropout with either of the studied medications, or in fact any medication for opioid use disorder. The aim of our present study was to assess the rather narrow view of this in looking at the current treatment climate in Nova Scotia with respect only to the few data points we had available in this secondary analysis. It is possible that in few cases, buprenorphine/naloxone is prescribed off-label for chronic pain, but this treatment has not been approved by guidelines and.

is unlikely to provide adequate pain relief for patients without opioid dependence or addiction.

## Conclusions

Our data suggests that methadone is a numerically superior medication for opioid use disorder when the metric of treatment retention is viewed in isolation, for our population in Nova Scotia. However, the results should be interpreted carefully considering the number of limitations of this study. There are social/accessibility, pharmacologic/safety, and patient preference factors which are also key in decision making when prescribing opioid agonist therapy. These must all be considered when deciding on which medication to initiate for a patient beginning a new treatment episode with OAT for opioid use disorder. This study should stimulate further research into this important area in addiction medicine.

## Data Availability

The datasets generated and/or analysed during the current study are not publicly available since it is part of the prescription monitoring program of the province of NS, Canada for controlled medications but are available from the corresponding author on reasonable request.

## References

[1] Rehm J, Goldman B, Fischer B, Kurdyak P, Gooch J (2016). Non-medical prescription opioid use, prescription opioid-related harms and public health in Canada: an update 5 years later. Can J Public Heal.

[2] Connery HS (2015). Medication-assisted treatment of opioid use disorder. Harv Rev Psychiatry.

[3] American Psychiatric Association. Diagnostic and statistical manual of mental disorders. Arlington. 2013. 10.1176/appi.books.9780890425596.744053.

[4] Bruneau J (2018). Management of opioid use disorders: a national clinical practice guideline. Can Med Assoc J.

[5] Fraser R, Mauger S, Gill K (2014). Utilizing buprenorphine/naloxone to treat illicit and prescription-opioid dependence. Neuropsychiatr Dis Treat.

[6] Ending old epidemics and addressing new outbreaks. Des Jarlais, D. C., Kerr, T., Carrieri, P., Feelemyer, J. & Arasteh, K. HIV infection among persons who inject drugs. Aids. 2016;30:815–25. 10.1097/QAD.0000000000001039 PMC4785082 26836787

[7] Calcaterra SL, et al. Methadone matters: what the United States can learn from the global effort to treat opioid addiction. J Gen Intern Med. 2019;23–26. 10.1007/s11606-018-4801-3. 10.1007/s11606-018-4801-3 PMC6544670 30729416

[8] Socias ME (2018). The OPTIMA study, buprenorphine/naloxone and methadone models of care for the treatment of prescription opioid use disorder: study design and rationale. Contemp Clin Trials.

[9] Saxon AJ, Commentary on Burns (2015). Retention in buprenorphine treatment. Addiction.

[10] Mattick R, Breen C, Kimber J, Davoli M. Buprenorphine maintenance versus placebo or methadone maintenance for opioid dependence ( review ) SUMMARY OF FINDINGS FOR THE MAIN COMPARISON. Cochrane Libr Syst Rev CD002207. 2014.

[11] Anderson-Baron J, et al. Principles, practice, and policy vacuums: policy actor views on provincial/territorial harm reduction policy in Canada. Int J Drug Policy. 2019;0–1. 10.1016/j.drugpo.2018.12.014. 10.1016/j.drugpo.2018.12.014 30711412

[12] Schleihauf E (2018). At-a-glance - concurrent monitoring of opioid prescribing practices and opioid-related deaths: the context in Nova Scotia, Canada. Heal Promot Chronic Dis Prev Canada.

[13] Bell J, Trinh L, Butler B, Randall D, Rubin G (2009). Comparing retention in treatment and mortality in people after initial entry to methadone and buprenorphine treatment. Addiction.

[14] Burns L (2015). A longitudinal comparison of retention in buprenorphine and methadone treatment for opioid dependence in New South Wales. Australia Addiction.

[15] Chan B (2021). Retention strategies for medications for opioid use disorder in adults: a rapid evidence review. J Addict Med.

[16] Eibl JK (2017). The effectiveness of telemedicine-delivered opioid agonist therapy in a supervised clinical setting. Drug Alcohol Depend.

[17] Ahmed S, Bhivandkar S, Lonergan BB, Suzuki J (2021). Microinduction of buprenorphine/naloxone: a review of the literature. Am J Addict.

[18] Randhawa PA, Brar R, Nolan S (2020). Buprenorphine–naloxone “microdosing”: an alternative induction approach for the treatment of opioid use disorder in the wake of North America’s increasingly potent illicit drug market. Can Med Assoc J.

[19] Lee SS, Humphreys KL, Flory K, Liu R, Glass K (2011). Prospective association of childhood attention-deficit/hyperactivity disorder (ADHD) and substance use and abuse/dependence: a meta-analytic review. Clin Psychol Rev.

[20] Kalbag AS, Levin FR (2005). Adult ADHD and substance abuse: diagnostic and treatment issues. Subst Use Misuse.

[21] Karlstad Ø, Furu K, Skurtveit S, Selmer R (2014). Prescribing of drugs for attention-deficit hyperactivity disorder in opioid maintenance treatment patients in Norway. Eur Addict Res.

[22] Becker SJ (2021). Effectiveness of medication for opioid use disorders in transition-age youth: a systematic review. J Subst Abus Treat.

[23] Medication-Assisted Treatment of Adolescents With Opioid Use Disorders (2016). Pediatrics.

[24] Harstad E, Levy S (2014). Attention-deficit/hyperactivity disorder and substance abuse. Pediatrics.

[25] Crowley D, Delargy I (2020). A national model of remote care for assessing and providing opioid agonist treatment during the COVID-19 pandemic: a report. Harm Reduct J.

[26] Cochran G, Bruneau J, Cox N, Gordon AJ (2020). Medication treatment for opioid use disorder and community pharmacy: expanding care during a national epidemic and global pandemic. Subst Abus.

[27] Oviedo-Joekes E, MacDonald S, Boissonneault C, Harper K (2021). Take home injectable opioids for opioid use disorder during and after the COVID-19 pandemic is in urgent need: a case study. Subst Abuse Treat Prev Policy.

